# A Modified Critical Nitrogen Dilution Curve for Winter Wheat to Diagnose Nitrogen Status Under Different Nitrogen and Irrigation Rates

**DOI:** 10.3389/fpls.2020.549636

**Published:** 2020-10-21

**Authors:** Yu Zhao, Pengfei Chen, Zhenhai Li, Raffaele Casa, Haikuan Feng, Guijun Yang, Wude Yang, Jianwen Wang, Xiaobin Xu

**Affiliations:** ^1^Key Laboratory of Quantitative Remote Sensing in Ministry of Agriculture and Rural Affairs, Beijing Research Center for Information Technology in Agriculture, Beijing, China; ^2^Agronomy College, Shanxi Agricultural University, Taigu, China; ^3^State Key Laboratory of Resources and Environmental Information System, Institute of Geographic Sciences and Natural Resources Research, Chinese Academy of Sciences, Beijing, China; ^4^Dipartimento di Scienze Agrarie e Forestali (DAFNE), Università della Tuscia, Viterbo, Italy

**Keywords:** water and nitrogen coupling effect, nitrogen nutrition index, plant water content, hierarchical linear model, modified critical nitrogen dilution curve

## Abstract

The accuracy of nitrogen (N) diagnosis is essential to improve N use efficiency. The standard critical N concentration (standard N_c_) dilution curves, an expression of the dynamics of N uptake and dry matter accumulation in plants, are widely used to diagnose the N status of crops. Several standard N_c_ dilution curves were proposed and validated for several crops, based on experiments involving different N fertilizer treatments. However, standard N_c_ dilution curves are affected by crop water status, e.g., resulting from differences in irrigation management. This paper aimed at developing a N diagnostic model under the coupling effect of irrigation and fertilizer managements. For this purpose, N_c_ dilution curves were developed under different irrigation rates. Additionally, plant water content (PWC), leaf water content (LWC), leaf area index (LAI), equivalent water thickness (EWT), and leaf area duration (LAD) were introduced into the model, to construct a modified N_c_ (mN_c_) dilution curve. The mN_c_ dilution curves were designed using the principle of hierarchical linear model (HLM), introducing aboveground dry biomass (AGB) as the first layer of information, whereas the second layer of information included the different agronomic variables (PWC, LWC, LAI, EWT, and LAD). The results showed that parameters “*a*” and “*b”* of the standard N_c_ dilution curves ranged from 5.17 to 6.52 and −0.69 to −0.38 respectively. Parameter “*a*” was easily affected by different management conditions. The performance of standard N_c_ dilution models obtained by the cross-validation method was worse than that of mN_c_ dilution models. The N_c_ dilution curve based on 4 years of data was described by the negative power equation N_c_ = 5.05 × AGB^–0.47^, with *R*^2^ and nRMSE of 0.63 and 0.21, respectively. The mN_c_ dilution curve considers different treatments and was represented by the equation mN_c_ = a×AGB^−*b*^, where *a* = 2.09 × PWC + *3.24, b* = −0.02 × LAI + 0.51, with *R*^2^ and nRMSE of 0.79 and 0.13, respectively. For winter wheat, C_3_ crop, there would be a few problems in using standard N_c_ dilution methods to guide field management, however, this study provides a reliable method for constructing mN_c_ dilution curves under different water and N fertilizer management. Due to the significant differences in hereditary, CO_2_ fixation efficiency and N metabolism pathways for C_3_ and C_4_ crops, the construction of mN_c_ dilution curve suitable for different N response mechanisms will be conducive to the sustainable N management in crop plants.

## Introduction

Nitrogen (N) is the element used in the largest quantity, as chemical fertilizer in agricultural production, causing environmental concerns worldwide (Reis et al., 2016). China is the largest user of N fertilizers in the world (FAO, 2015). Over-application of N fertilizers not only wastes tremendous resources, but also threatens the health of the environment (Wei et al., 2018). Rapid and accurate diagnosis of the N status of crops is needed to improve its management and achieve sustainable agricultural development goals in China and worldwide, given the usually low efficiency of use of this element in most cropping systems (Cassman, 1999). How to improve the accurately of nitrogen diagnosis is one of the keys to alleviating the environmental problems caused by an unreasonable N use.

The standard critical N (standard N_c_) dilution curve has been widely used to diagnose the N status of crops (Justes et al., 1994; Lemaire and Meynard, 1997). The standard N_c_ is defined as the minimum N concentration required to achieve maximum growth at a certain aboveground dry biomass (AGB) accumulation, which is expressed as N_c_ = *a* × AGB^–b^, where the parameter “*a”* represents standard N_c_ for per 1 Mg AGB ha^–1^, and parameter *“b”* is a statistical parameter governing the slope of the relationship (Lemaire and Meynard, 1997). Different approaches, in addition to the original one based on AGB (N_c_-AGB) (Justes et al., 1994), have been developed to derive the standard N_c_ dilution curve, e.g., using the development stages (N_c_-stage) (Zhao et al., 2014), or the leaf area index (LAI) (Ata-Ul-Karim et al., 2014b). As a practical tool for N status diagnosis, the N Nutrition Index (NNI) was proposed, based on the standard N_c_ model (Lemaire and Meynard, 1997) and has become widely used on a range of different crops (Dordas, 2011; Caviglia et al., 2014; Huang et al., 2015). The NNI, as established using agronomic data or remote sensing data, has been applied to diagnose N status, recommend appropriate N fertilizer inputs, predict the grain protein content, and estimate yields (Xue and Yang, 2008; Gaju et al., 2014; Qu et al., 2017). The standard N_c_ dilution model for winter wheat was first established by Justes et al. (1994) and has been extensively applied in many studies. However, the standard N_c_ dilution model is strongly influenced by climate, topography, and agronomic management in different regions (Ziadi et al., 2010; Yue et al., 2012; Huang et al., 2018). Besides, the shape of the standard N_c_ dilution curve is determined by allometric relationships between N concentration and structural growth and metabolism, which could vary across genotypes. Greenwood et al. (1990) established independent standard N_c_ dilution models for species with different carbon cycles. One model was for C_3_ species (N_c_ = 5.7AGB^–^*^0.5^*: tall fescue, lucerne, potato, wheat, rape, and cabbage) and another was for C_4_ plants (N_c_ = 4.1 × AGB^–^*^0.5^*: sorghum and maize). Dry matter partitioning among different plant organs affects the shape of the standard N_c_ curve (Kage et al., 2002). Therefore the introduction of correction factors taking into account these effects has been proposed (Ratjen and Kage, 2016). Previous models have taken into account differences in N_c_ dilution curves based on stem biomass, leaf biomass, LAI, and spike biomass, although differences due to environmental factors, such as crop water availability have been less investigated (Ata-Ul-Karim et al., 2014a, b; Yao et al., 2014; Zhao et al., 2016). Errecart et al. (2014) have shown that the critical N dilution curve for tall fescue in water deficit conditions was lower than the critical curve in irrigated conditions. Their conclusions postulated that water limited plants should have an intrinsic lower critical N concentration (for a similar biomass) than well-watered plants. More recently, Kunrath et al. (2018) analyzed more deeply N-water interactions for tall fescue and alfalfa by using N dilution curves. They concluded that (i) water deficit had a strong effect on both mineral N availability for grass and N biological fixation for alfalfa; (ii) the reduction of water transpiration efficiency of both grass and legume crop was strictly proportional the reduction of their N status; and (iii) the ratio N uptake/transpiration was a relevant estimator of the effect of water-N interactions.

Practically, the effect of irrigation and fertilization on standard N_c_ dilution curves was considered by constructing a piecewise N_c_ model under different irrigation conditions (Rong and Nong, 2017). Pandey et al. (2000) analyzed water deficit effects on shoot growth, N uptake and water extraction with varying level of N supply. Their results showed that crops differ in their ability to maintain AGB at different levels of water deficit and N supply. Therefore, it seems worth considering the effect of water status on the standard N_c_ dilution curve if different irrigation regimes are used. Irrigation not only affects the availability of soil nitrogen, but also affects N uptake, transport, and assimilation. Insufficient water supply limits the efficient use of N, whereas excessive water supply results in N leaching, increased N loss, and reduced crop yields. Numerous studies have shown that the interacting effect of water and N is a complex problem that affects crop growth and development (Wang et al., 2015, 2018). Adequate N supply is essential to utilize the benefits of additional water from rainfall and irrigation. Conversely, adequate water supply is required to use the benefits of N fertilization. The parameters *“a”* and *“b,”* defined above, are therefore affected by both irrigation and N rates. A significant issue is how to modify the standard N_c_ dilution curve, in order to include the information on water status, e.g., considering irrigation volumes.

The hierarchical linear model (HLM) has been introduced for the analysis of hierarchically structured data. It is a statistically sound methodology with regression models that explicitly take into account variability at different levels (Kuo et al., 2000; Chakraborty et al., 2018). The model provides an analytical method that resolves the complex interactions of nesting in social, economic, and environmental contexts (Hebblewhite and Merrill, 2008). Li et al. (2020) used the HLM model to determine the protein content of grain under diverse environmental conditions and obtained a higher accuracy than linear regression models.

Leaf or stem water potential (ψ) (Meron et al., 1987), crop water stress index (CWSI) (Idso et al., 1981), relative leaf water content (Bowman, 1989; Hunt and Rock, 1989; Ceccato et al., 2001) and other indicators are often used to characterize water deficit status of plants. Pre-dawn or midday leaf water potential measurements have been widely used to assess plant water status (Meron et al., 1987; Donovan et al., 2001), but they are incredibly impractical. There has been much interest in the CWSI as a potential tool for water stress monitoring from remote platforms (Cohen et al., 2005; Veysi et al., 2017). However, it requires thermal infrared data, which are typically acquired at a lower resolution than optical data. On the other hand, leaf (LWC) or plant water content (PWC) can be obtained more easily from optical remote sensing platforms (Ceccato et al., 2001).

Our objectives in this study were: (i) to investigate the effects of different irrigation treatments on the standard N_c_ dilution model for winter wheat, (ii) to construct modified N_c_ (mN_c_) dilution models that include different agronomic variables, i.e., PWC, LWC, LAI, equivalent water thickness (EWT), and leaf area duration (LAD), as correction factors; (iii) to validate the standard N_c_ dilution model and the newly proposed mN_c_ dilution models using independent data.

## Materials and Methods

### Experimental Design

**FIGURE 1:**
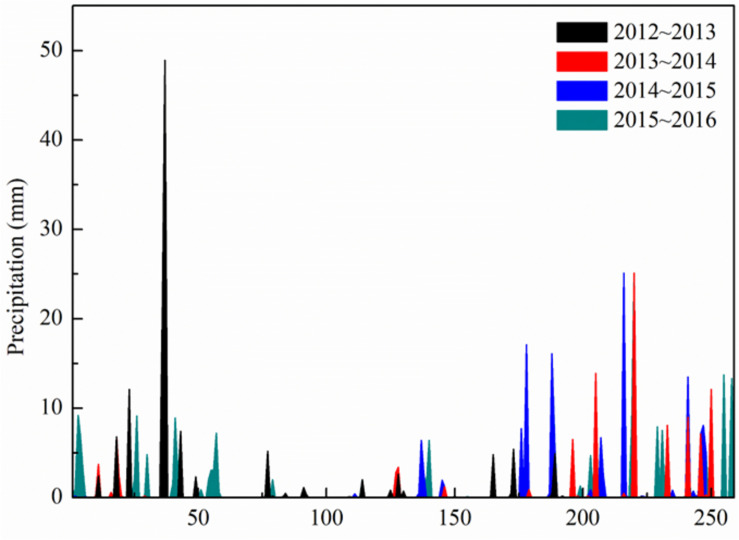
Precipitation at the experimental site during each growing season.

Experiments were carried out during the 2013 to 2016 growing seasons at the Xiaotangshan Experimental site (40°11′31″∼40°11′18″N, 116°26′10″∼117°27′05″E) in Beijing, China. The soil was classified as heavy loam (FAO, 2015) with 15.3∼19.1 mg kg^–1^ organic matter, 8.21 ∼ 41.71 mg kg^–1^ nitrate-N, 0.45 ∼ 8.20 mg kg^–1^ ammonium-N, 60.58 ∼ 109.61 mg kg^–1^ available potassium, and 3.14 ∼ 21.18 mg kg^–1^ available phosphorus at 0 ∼ 30 cm soil depth. Precipitation data were obtained from the European Center for Medium-Range Weather Forecasts (ECMWF)^1^ (Figure 1). The ERA-Interim data at 0.125° resolution has been proved to be reliable and applied to the prediction of crop production (Poli et al., 2010; Xu et al., 2020).

Data were obtained from four winter wheat growing seasons, as summarized in Table 1. Experiment 1 was conducted during the 2012 ∼ 2013 growing season, using a randomized complete block design with two replicates of two factors: N fertilizer and wheat verities [Nongda211(P1), Zhongmai 175 (P2), Jing9843 (P3) and Zhongyou 206(P4)]. The N fertilizer treatments in experiment 1 were as follows: (i) 0 kg N ha^–1^ (N1); (ii) 110 kg N ha^–1^ (N2); (iii) 220 kg N ha^–1^ (N3), and (iv) 440 kg N ha^–1^ (N4). Experiments 2 and 3 were conducted during the 2013 ∼ 2014 and 2014 ∼ 2015 growing seasons, using an orthogonal design of *L*_16_(*3*^4^), with three replicates and testing three factors: N fertilizer rates, wheat varieties, and irrigation rates. The N fertilizer treatments were as follows: (i) 0 kg N ha^–1^ (N1); (ii) 90 kg N ha^–1^ (N2); (iii) 180 kg N ha^–1^ (N3), and (iv) 270 kg N ha^–1^ (N4). Irrigation rates in experiment 2 were 0 mm (W0), 146 mm (W1), and 292 mm (W2). Irrigation rates in experiment 3 included 0 mm (W0), 192 mm (W1), and 384 mm (W2). Experiment 4 was conducted during the 2015 ∼ 2016 growing season, using a randomized complete block design with three replicates. N fertilizer was applied with the following rates: (i) 0 kg N ha^–1^ (N1), (ii) 90 kg N ha^–1^ (N2), (iii) 180 kg N ha^–1^ (N3), and (iv) 270 kg N ha^–1^ (N4). Wheat varieties included Lunxuan 167 and Jingdong 18. Fertilizers included urea (46% of N), superphosphate (12% of P_2_O_5_), and potassium sulfate (50% of KCl). The N fertilizer was applied in two splits for all experiments: 50% as a basal application before sowing and 50% at the jointing stage. For all treatments, sufficient P (45 kg P_2_O_5_ ha^–1^) and K (75 kg KCl ha^–1^) fertilizers were incorporated into the soil before sowing. Plot designs for the different growing seasons are inconsistent with Xu et al. (2020).

**TABLE 1:** Experimental field information of each growing season.

Experiment^*a*^	Season	Sowing date	Sampling period	Zadoks stage	No.^*a*^
Exp1	2012 ∼ 2013	27th September	Jointing Booting Anthesis Filling	31 47 65 75	48 48 48 48
Exp2	2013 ∼ 2014	4th October	Jointing Booting Anthesis Filling	31 45 65 75	48 48 48 48
Exp3	2014 ∼ 2015	7th October	Jointing Booting Anthesis Filling	31 47 65 75	48 48 48 48
Exp4	2015 ∼ 2016	27th September	Jointing Booting Anthesis Filling	31 47 65 75	24 24 24 24

*^*a*^ Exp and No. represent the experiment code (see text) and the number of samples, respectively.*

### Plant Sampling and Analysis

Twenty tillers of winter wheat plants per experimental plot were randomly sampled at jointing, booting, anthesis and filling stages, i.e., respectively, Zadoks stage (ZS) 31, 47, 65, 75 (Zadoks et al., 1974), to determine AGB, PNC, PWC, LWC, LAI, EWT, and LAD. The number of samples collected in each experiment is reported in Table 1. The wheat samples collected were sealed in a plastic bag. Upon returning from the field, leaves and stems were separated and weighed. Samples were then packed in paper bags, green-killed for 30 min at 105°C, and dried at 80°C to a constant weight. Fresh AGB and dry AGB were calculated according to the number of winter wheat stems per unit area during each growth period. The formula for calculating PWC and LWC was as follows:

(1) PWC=(AGB-freshAGB)/AGBhfres

(2) LWC=(AGB-l-freshAGB)l/AGBl-fresh

Where AGB_fresh_, AGB, AGB_l–fresh_, and AGB_l_ represent the fresh plant aboveground biomass, aboveground dry biomass, fresh leaf biomass and dry leaf biomass, respectively.

The N content of leaves, stems, and spikes were determined using the standard Kjeldahl method. PNC was calculated using the following formula:

PNC(%)=(N×lAGB+lN×sAGB+sN×p

(3) AGB)p/(AGB+lAGB+sAGB)e

Where N_l_, N_s_, and N_p_ represent leaf N concentration, stem N concentration, and panicles N concentration, respectively. AGB_l_, AGB_s_, and AGB_p_ represent dry leaf biomass, dry stem biomass, and dry panicles biomass, respectively.

Leaf area was measured with a laser leaf area meter (CI-203, CID Inc., Camas, WA, United States), and the calculation formula of LAI is as follows:

(4) LAI=LA/mn×T/A×10000(n=20)

Where LA_m_, n, T, and *A* represent the leaf area of collected wheat, the number of collected tillers, the number of tillers in the survey area and the size of the survey area, respectively.

The calculation formula of leaf EWT and LAD is as follows:

(5) EWT=(AGB-l-freshAGB)l×LAm

(6) LAD=(LAI-2LAI)1/(T-2T)1

Where T and LAI represent time and leaf area index.

### Critical N (N_c_) Concentration Dilution Curve

#### Standard N_c_ Model

**FIGURE 2:**
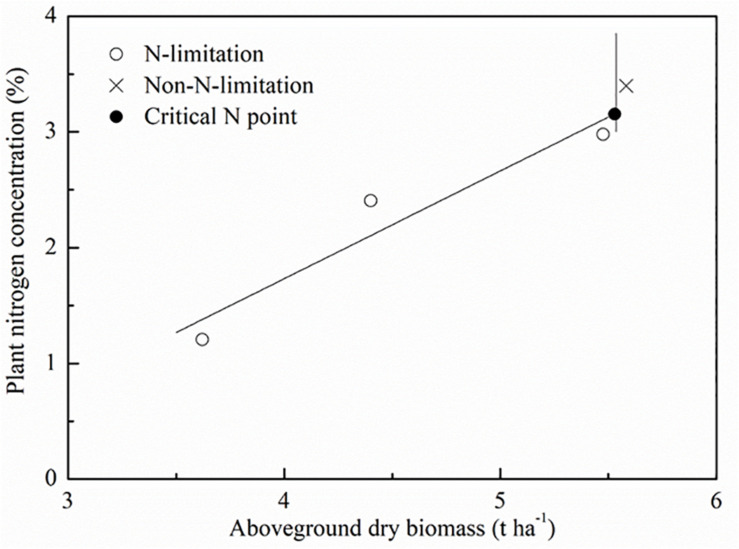
Schematic diagram of standard critical N (N_c_) concentration point selection. The symbols (O) and (X) represent the data points for N-limiting treatments and N-limiting treatments. The symbol (•) represents the calculated standard N_c_ points.

With the standard N_c_ dilution curve approach, it is essential to determine standard N_c_ points at which N fertilizer neither limits plant growth nor enhances it. We used the same computational procedures followed by Justes et al. (1994). A standard N_c_ point was determined as follows: (a) the AGB and PNC under different applied N rates were compared through the least significant difference test (LSD) at the 95% level of significance (SPSS Inc., Chicago. IL, United States). The N-limiting treatment was defined as a treatment in which an additional N fertilizer application led to a statistically significant increase in the AGB. The non-N-limiting treatment was identified as a treatment in which an additional N fertilizer application did not lead to a significant increase in the AGB, but resulted in a significant increase in the PNC. The AGB and PNC were used to determine whether N treatments limited crop growth. A simple linear regression was utilized to fit data from the N-limiting treatments. For each sampling period, a critical point was defined as follows: (a) the data of AGB and PNC were used to identify if a N treatment limited the growth of the crop; (b) a simple linear regression was used to fit data from the N-limiting treatments (the oblique line); (c) The maximum AGB was calculated with data from the non-N-limiting treatments as the average of the observed data (the vertical line); (d) the N_c_ point corresponded to the ordinate of the intersection point of the oblique and vertical lines. Figure 2 is a schematic diagram of standard N_c_ point selection procedure. The N_c_ points were then fitted using a power regression equation to construct the standard N_c_ curve. The standard N_c_ dilution curve is calculated as:

(7) N=ca×AGBb-

Where “*a*” and “*b*” are parameters obtained by calibrating the standard N_c_ dilution curve.

#### Modified N_c_ (mN_c_) Dilution Curve Under Different Irrigation and N Rates

In the standard approach, parameters “*a*” and “*b*” are considered as constant, regardless of different irrigation and fertilizer treatments, although it is known that these factors influence the parameters (Errecart et al., 2014). We propose here a mN_c_ dilution curve that allows parameters “*a*” and “*b*” vary under different irrigation and fertilizer treatments, for a better characterization of the N diagnosis. Other agronomic variable, i.e., PWC, LWC, LAI, EWT, and LAD, are assessed to improve the standard N_c_ dilution curve calculation.

The hierarchical linear model (HLM) was introduced for the analysis of nested structured data (Lininger et al., 2015). It is a statistically sound methodology with regression models that explicitly consider variability at different levels. We followed the common approach suggested in the literature (Lininger et al., 2015) to build HLMs, considering the sources of variation at two levels. At level 1, mN_c_ varies over time as a negative power function of a specific irrigation rate. The change function describes the trajectory of standard N_c_ over AGB and is characterized by a set of specific change parameters. At level 2, these specific change parameters are viewed as varying across irrigation treatments, possibly a function of different agronomic variables. For the mN_c_ dilution curve there are two additional independent variables passed to the next level in the HLM (Level-2) that again considered parameter “*a*” and “*b*” as a function of agronomic variables. Therefore, the parameters to be estimated for mN_c_ dilution curve are the four parameters in Level-2 (β_1_, β_2_, β_3_, and β_4_). The HLM model parameters were obtained using optimization algorithm in Matlab 2016 (Mathworks, Inc., Natick, MA, United States) programming. The mN_c_ dilution curve is described as follows:

(8) Level1:mN=ca×AGBb-

(9) Level2:a=β×1f(AV)1+β2

(10) b=β×3f(AV)2+β4

**FIGURE 3:**
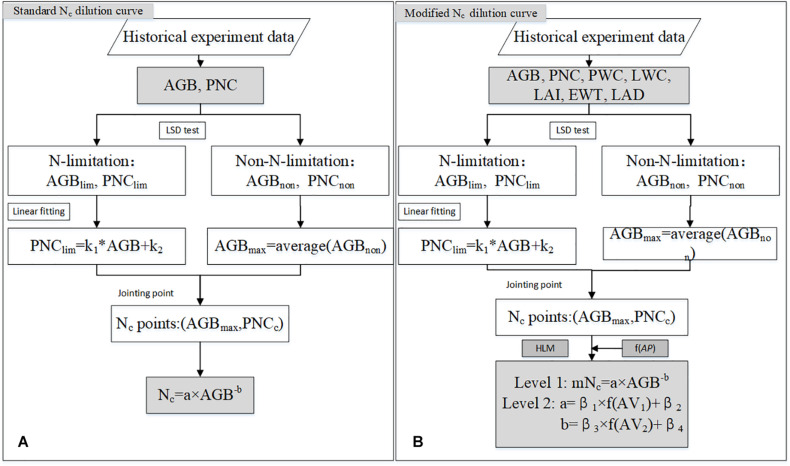
Flow chart for standard N_c_ dilution model **(A)** and the mN_c_ dilution model **(B)**. AGB_lim_ and PNC_lim_ represent AGB and PNC corresponding to N limitation plots, respectively. AGB_non_ and PNC_non_ represent AGB and PNC corresponding to non-N -limitation plots, respectively. The k_1_ and k_2_ were parameters obtained by calibrating PNC_lim_ with AGB_lim_.

Where β_1_, β_2_, β_3_, and β_4_ are the parameters to be estimated of the mN_c_ model, respectively. β_1_ and β_3_ represent slopes of each agronomic variable. β_1_ and β_3_ represent the intercept. AC represents one of the different agronomic variables tested. AC_1_ and AC_2_ are one of five agronomic variables (i.e., PWC, LWC, LAI, EWT or LAD) randomly selected, respectively, including 25 combinations. Figure 3 shows a diagram of the methodologies used to obtain the standard N_c_ and the mN_c_ models.

### Nitrogen Nutrition Index (NNI)

NNI is the ratio of the actual plant N concentration to the mN_c_ value for the corresponding biomass value. In order to verify the N diagnostic function of mN_c_ dilution curve, the corresponding prediction NNI was calculated as:

(11) $$\text{NNI}=\text{PNC}/\mathrm{mN}{}_{c}$$

where NNI, PNC, and mN_c_ represent nitrogen nutrition index, plant N concentration and predicted critical nitrogen concentration points using the mN_c_ method, respectively.

### Model Evaluation

To test whether there was a significant difference in the regression relationships under different management conditions, we examined the difference of regression coefficients using the *F*-test (SPSS Inc., Chicago, IL, United States). We evaluated model performance using the determination coefficient (*R*^2^) and the normalized root mean squared error (nRMSE). These were calculated as follows:

(12) $$R^{2}=1-\frac{\sum_{i=1}^{n}\left(Y_{i}-Y_{i}^{\prime }\right)/\left(n-p-1\right)}{\sum_{i=1}^{n}{\left(Y_{i}-Y_{i}^{\prime }\right)}^{2}/\left(n-1\right)}$$

(13) $$\text{RMSE}=\sqrt{\frac{1}{n}\,\sum_{i=1}^{n}{\left(Y_{i}-Y_{i}^{\prime }\right)}^{2}}$$

(14) nRMSE=RMSE/mean⁢(Y)

Where, *n*, $Y_{i}^{\prime }$, *Y*_*i*_, and *p* represented the number of samples, predicted value, measured value and the number of independent variables, respectively.

Akaike information criterion (AIC) was used to find out whether the mN_c_ dilution curve, in which more parameters are included, performed better than the standard N_c_ dilution curve. The AIC value is closely related to L and K value. Among them, the smaller the K value is, the more concise the model is, and the larger the L value is, the more accurate the model is. The general equation is (Bartholomeus, 1987):

(15) AIC=(-2⁢L+2⁢K)/n

(16) L=(n/2)×ln⁢(2×PI)-(n/2)×ln⁢(SSe/n)-n/2

Where L and K represent log-likelihood function and the numbers of estimated parameters, respectively. The PI, Sse and n represent circular constant, residual sum of squares and the samples number, respectively.

## Results

### Standard N_c_ Dilution Curve for Winter Wheat

**FIGURE 4:**
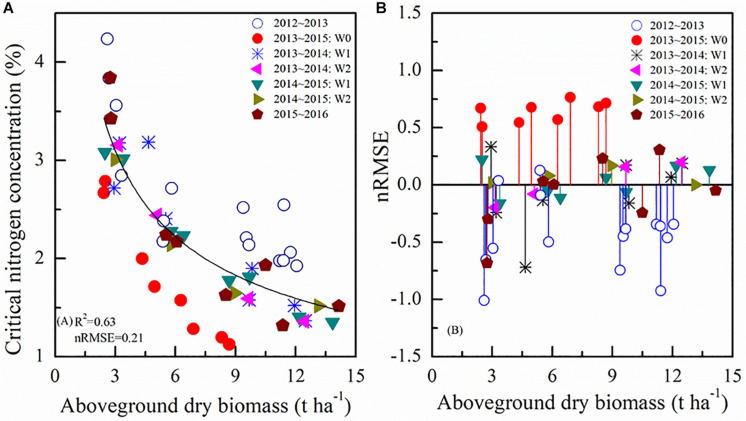
Standard N_c_ dilution curve **(A)** during different growing seasons and nRMSE values using standard N_c_ method **(B)**. 2012∼2013 and 2015∼2016 represent different winter wheat growing seasons. W represents irrigation treatments.

According to the standard N_c_ dilution theory, the N_c_ dilution curve divides the data points into three categories: N-limited status below the curve, N-excess states above the curve, and N optimum close to the curve. The average standard N_c_ dilution curve based on all the data pooled together could be characterized by the negative power equation N_c_ = 5.05×AGB^–^*^0.47^*, with *R*^2^ and nRMSE, of 0.63 and 0.21. However, using the standard N_c_ dilution curve, there was a large variability depending on the year and water availability conditions (Figure 4A). Additionally, it is known that in water limited conditions (W0) the N uptake is also affected (Kunrath et al., 2018), thus the estimation of standard N_c_ dilution curve is influenced by uncertainty and possibly underestimated.

Figure 4B represents the nRMSE between the value of selected points using the standard N_c_ method and the predicted N_c_ value using the average standard N_c_ dilution curve. It shows greater nRMSE values for the most diverse irrigation conditions. The average standard N_c_ dilution curve overestimated or underestimated the data from 2013 to 2015 *W*0 and 2012 to 2013 respectively, greatly impacting the N status assessment, needed e.g., to carry out reasonable N applications in the field.

**FIGURE 5:**
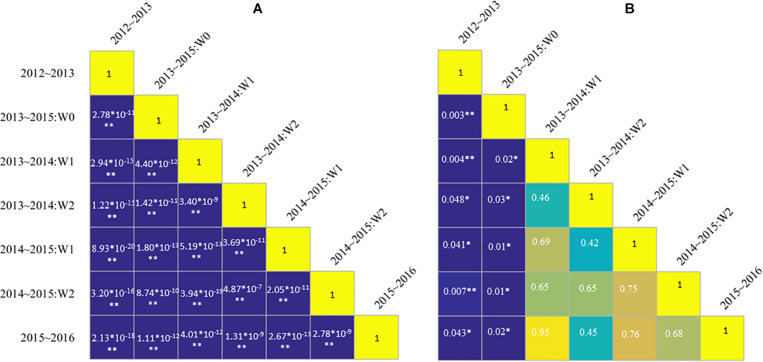
Matrix of significant differences among parameters “*a*” **(A)** and “b” **(B)** of standard N_c_ dilution curves obtained separately for the different experiments. A significant level *P* < 0.05 and *P* < 0.01 is indicated by the symbol * and **, respectively. W represents irrigation treatments.

Instead, when specific N_c_ dilution curves were developed for the different treatments, i.e., developing different models for each experiment, significant differences in the parameters “*a*” and “*b*” were found (Figure 5). The parameter “*a*” ranged from 5.17 to 6.52, whereas parameter “*b*” ranged from −0.69 to −0.38 (Table 2). Standard N_c_ models based on different water management conditions showed better performances, with ranges of *R*^2^ and nRMSE respectively of 0.78 to 0.96 and 0.03 to 0.20. The value of parameter “*a*,” i.e., the N_c_ for an AGB of 1 Mg ha^–1^, obtained from the 2012–2013 and from the 2013–2015 non-irrigated data (W0) data was significantly different from that derived from all the other experiments. In general, whereas parameter “*a*” was significantly different among the experiments, there was no significant difference in parameter “*b.*”

**TABLE 2:** Parameters of the standard N_c_ dilution curve for winter wheat obtained in the different experiments.

Treatments	Irrigation rates	*a*	*b*	*R*^2^	nRMSE
2012∼2013	187 mm	5.17	−0.38	0.78	0.12
2013∼2015: W0	25 mm	5.19	−0.69	0.96	0.05
2013∼2014: W1	171 mm	5.86	−0.54	0.86	0.14
2013∼2014: W2	317 mm	6.52	−0.62	0.95	0.03
2014∼2015: W1	217 mm	5.35	−0.51	0.85	0.04
2014∼2015: W2	409 mm	5.30	−0.48	0.64	0.20
2015∼2016	180 mm	6.33	−0.58	0.91	0.09

*W represents different irrigation treatments.*

### Agronomic Variables of Winter Wheat Under Different N Fertilizer and Irrigation Treatments

In order to further analyze the coupled effect of irrigation and fertilizer management on different agricultural variables, we examined their differences in different growth periods under irrigation and fertilizer management, using the LSD methodology. The results of the differences (*P*-values) between separate management on the agronomic variables considered are reported in Table 3. There was significant (*P* < 0.05) influence of management on LAI at jointing, booting and anthesis stages under different irrigation and N rates, but not in the filling stage. There was no significant influence of irrigation or fertilization treatments on PWC, LWC, EWT, and LAD.

**TABLE 3:** *P*-value between irrigation and N rates on PWC, LWC, LAI, EWT, and LAD.

	Jointing stage *ZS* = 31	Booting stage *ZS* = 45	Anthesis stage *ZS* = 65	Filling stage *ZS* = 75
PWC	0.44	0.74	0.65	0.99
LWC	0.56	0.99	0.95	0.98
LAI	2.71 × 10^–2^*	0.04*	3.00 × 10^–3^*	0.76
EWT	0.99	0.98	0.99	0.80
LAD	0.53	0.17	0.58	0.68

A significant level *P* < 0.05 is indicated by the symbol*. PWC, plant water content; LWC, leaf water content; LAI, leaf area index; EWT, equivalent water thickness; LAD, leaf area duration; ZS, Zadoks stage.

### The mN_c_ Dilution Curve

**FIGURE 6:**
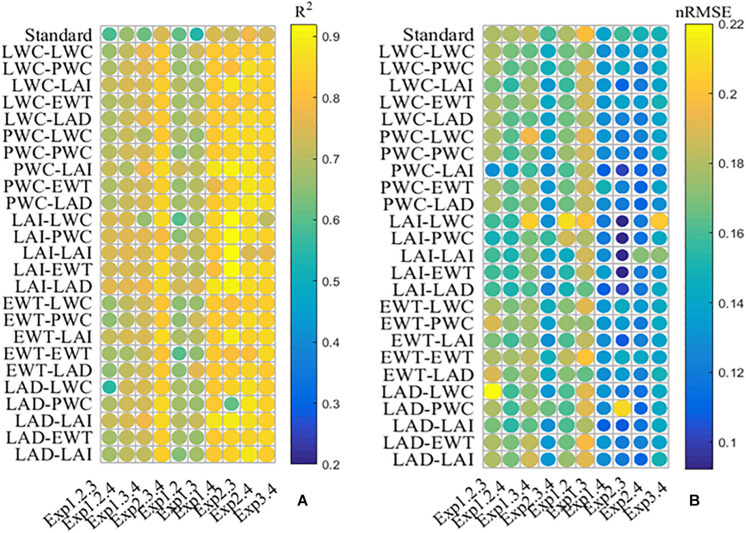
The determination coefficient **(A)** and the normalized root mean squared error **(B)** between all possible mN_c_ dilution models. The values of ordinates are composed of two agronomic parameters (API-AP2) (see Eqs. 9 and 10). API and API represent coefficients “*a*” and “*b*,” respectively.

A mN_c_ dilution model was constructed employing agronomic variables as an additional layer of information for the different treatments. Figure 6 presents *R*^2^ and nRMSE of the mN_c_ dilution curve using calibrated sets. Compared to the standard method, all the mN_c_ dilution models obtained better performances. During Exp1.3.4, Exp2.3.4, Exp1.4, and Exp2.4, the mN_c_ models using PWC and LAI, representing parameter “*a*” and “*b*,” obtained best performance with *R*^2^ of 0.78, 0.86, 0.88, and 0.88 and nRMSE of 0.16, 0.12, 0.11, and 0.11, respectively. During Exp1.2.3, Exp1.2.4, and Exp1.3, the mN_c_ models using LAI and LAD, representing parameter “*a*” and “*b*,” obtained best performance with *R*^2^ of 0.76, 0.76, and 0.76 and nRMSE of 0.16, 0.15, and 0.16, respectively. During Exp1.2 and Exp2.3, the mN_c_ models using LAI and LAI, representing parameter “*a*” and “*b*,” obtained best performance with *R*^2^ of 0.75 and 0.92 and nRMSE of 0.15 and 0.09, respectively.

We selected the model using PWC and LAI, representing parameter “*a*” and “*b*” respectively, for further analysis. Although this model did not have the highest *R*^2^ across all experiments, the nRMSE was always quite low. The relationship between measured mN_c_ and predicted mN_c_ using validation sets is shown in Supplementary Figure S1. The mN_c_ dilution models from the corresponding calibration sets, achieved a satisfactory performance with nRMSE range of 0.11 to 0.23 on the validation sets.

### Nitrogen Nutrition Index Based on mN_c_ Dilution Curve

**FIGURE 7:**
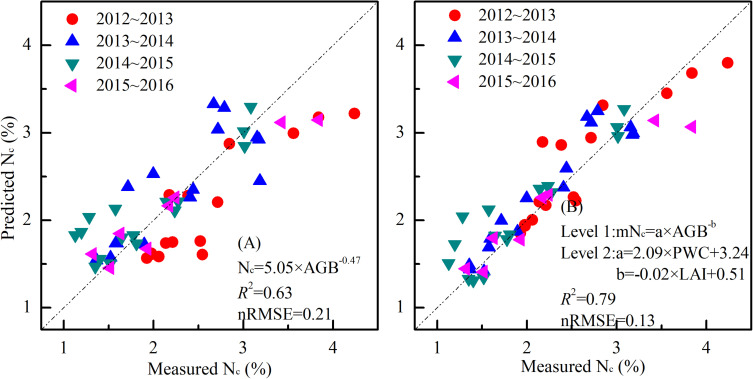
Relationships between measured standard N_c_ and predicted standard N_c_ using a unique standard N_c_ method **(A)** mN_c_ method **(B)** for all the experiments.

In order to have a unique model for all the experiments and to evaluate its effectiveness in N diagnosis, we constructed the standard N_c_ dilution model and the mN_c_ dilution model with all the data and compared their performance (Figure 7). The standard N_c_ dilution model was expressed as N_c_ = 5.05 × AGB^–0.47^, with *R*^2^ and nRMSE of 0.63 and 0.21. The mN_c_ dilution curve was represented by the equation mN_c_ = a×AGB^−*b*^, where *a* = 2.09 × PWC + 3.24, b = −0.02^∗^LAI + 0.51, with *R*^2^ and nRMSE of 0.79 and 0.13, respectively.

**FIGURE 8:**
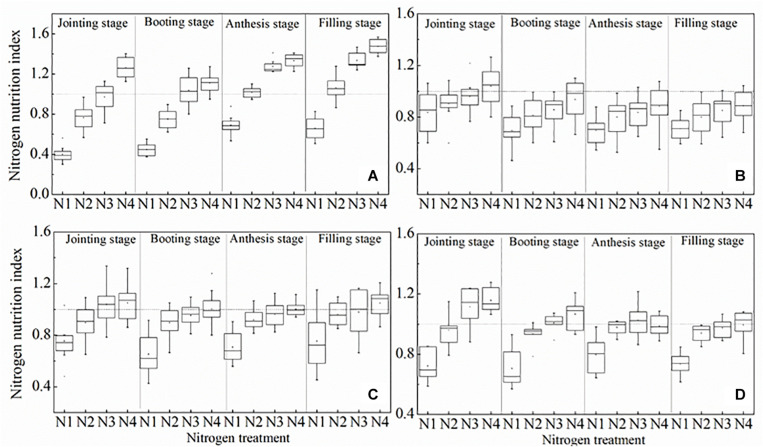
Changes in the N nutrition index on mN_c_ dilution curve at different growth period. **(A)** 2012 ∼ 2013, **(B)** 2013 ∼ 2014, **(C)** 2014 ∼ 2015, **(D)** 2015 ∼ 2016.

Our results showed that NNI decreased with the declining rate of N application, and NNI was significantly different at different growth periods (Figure 8). NNI values were less than 1 for N1 treatments for both the calibration and the validation set. NNI values of N2 treatments and N3 treatments were close to 1, indicating that these treatments were optimal for winter wheat. NNI values of N4 treatments were almost always greater than 1. These results illustrate that NNI based on the mN_c_ dilution curve can be used as a more robust diagnostic tool for N status of winter wheat as compared to the standard methodology.

Since the mN_c_ dilution model, employs more parameters than the standard methodology, the AIC was calculated to assess differences in terms of how concise the models are considering the number of parameters (Table 4). The results showed that the AIC index of mN_c_ dilution model was lower than standard N_c_ dilution model, even after additional input variables were added in the mN_c_ dilution model, which further confirmed that the mN_c_ dilution model is a more efficient model in addition to being a better predictor for different irrigation rates than the standard N_c_ dilution model.

**TABLE 4:** Predicting AIC value of N_c_ and mN_c_ for winter wheat.

	SSe	K	L	AIC
N_c_	19.25	−186.86	2	3.43
mN_c_	9.311	−146.92	4	2.74

## Discussion

### Critical Nitrogen Dilution Curves in Wheat

**FIGURE 9:**
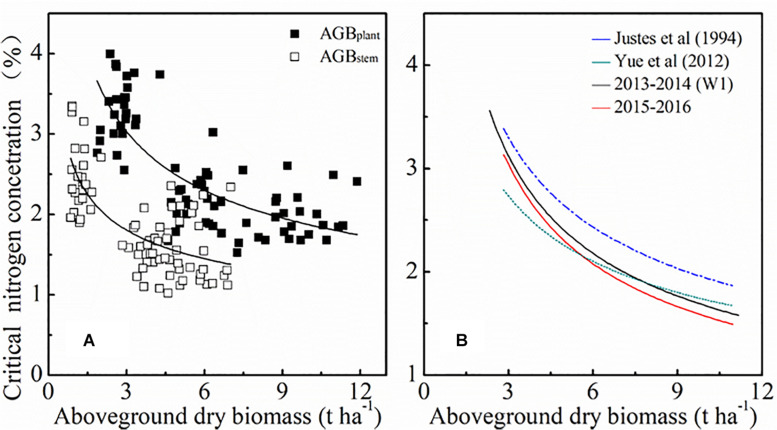
Comparison of standard N_c_ dilution curves for different plant organs **(A)** and produced by different studies **(B)**. Blue line represents the standard N_c_ dilution curve of Justes et al. (1994) (N_c_=535×AGB^0.44^). Brown line represents the standard N_c_ dilution curve of Yue et al. (2012) (N_c_=4.15×AGB^–^*^0.38^*). Black line represents the standard N_c_ dilution curve of the current study [2013 ∼ 2014 (*Wl*): N_c_=5.35×AGB^–0.53^]. Red line represents the standard N_c_ dilution curve of the current study (2014 ∼ 2015: N_c_=5.30×AGB^–0.50^).

The standard N_c_ dilution curve of winter wheat was expressed using the negative power relationship between AGB and PNC in this paper. The developing morphology of tissues and organs, the N proportion and the internal physiological mechanisms of winter wheat at different growth stages may result in declining PNC (Kage et al., 2002). Figure 9 showed the contribution of dry stem matter toward total plant dry matter is significantly higher than that of leaf dry matter. The main components of winter wheat stems are carbohydrates such as cellulose, hemicellulose, and lignin, which results in a lower N concentration in stems. The stem standard N_c_ dilution curve and the plant standard N_c_ dilution curve are shown in Figure 9A. Similar results were achieved by Ata-Ul-Karim et al. (2014a), when they analyzed the crop model of rice stems and plant standard N_c_. Future studies will consider the ratio of carbon and N and material distribution in plants to further explore the standard N_c_ dilution curve.

The parameters of the standard N_c_ dilution curve under local irrigation management established in this paper were different from those of predecessors, as shown in Figure 9B. The standard N_c_ dilution curves in the current study were lower than that of Justes et al. (1994). The reasons for this discrepancy could be related to a relatively longer growing period and more favorable weather conditions (lower spring temperature and more rainfall) in Europe. Higher temperatures will reduce the growth days, which give winter wheat less time to accumulate N in AGB. However, the standard N_c_ dilution curves in this study were similar to the curve constructed by Yue et al. (2012). In that study, the two experimental sites were both located in the North China Plain which has a continental monsoon climate. Therefore, climatic conditions may possibly explain some differences in standard N_c_ dilution curves. The standard N_c_ dilution curve in this study was constructed for different irrigation rates, varieties, and N rates. In future studies, more dataset from different ecological sites should be introduced to improve the universality of the model.

### The mN_c_ Dilution Curve for Winter Wheat Based on HLM Model

Figure 3A showed that the standard N_c_ value at the same biomass level was different under different treatments. In this study, the effects of irrigation and N rates on standard N_c_ dilution curves were analyzed using rain-fed, moderate, and excessive irrigation treatments, but the effects of water on the internal structure and physiological mechanisms of crops were not investigated. Subsequent studies may further explore the duration of irrigation, irrigation frequency, and irrigation gradient. Based on the above results, standard N_c_ value was affected not only by N rates, but also by irrigation rates. The standard N_c_ dilution model (Figure 4B) overestimated N_c_ value for the 2013 ∼ 2014 rainfed (W0) condition, but underestimated predicted N_c_ value in 2012 ∼ 2013. The N_c_ dilution curves constructed in this paper under different irrigation conditions were all a negative power type, but there were some differences among the parameters. Parameter “*a”* and “*b”* under different treatment were dissimilar. As shown in Figure 5B, the parameter “*a*” of different management had a significant difference. There was no significant difference for parameter “*b*” under most treatments. Therefore, different management was shown to affect mainly parameter “*a*”. The reason for the above results maybe that parameter “*a*” represents standard N_c_ for 1 t AGB ha^–1^. This biomass value might be occurring at different stages for different irrigation treatments. Instead, parameter *“b”* is a statistical parameter governing the slope of the relationship (Ziadi et al., 2008). While an appropriate level of irrigation is conducive to improving N absorption rate, excessive irrigation accelerates N leaching and reduces fertilizer efficiency (Sandhu et al., 2000). Therefore, this paper argues that the standard N_c_ dilution curve could be inaccurate for field management under the coupling effect of water and N fertilizer. The validation results in this paper also proved these problems.

This paper proposed a standard mN_c_ dilution model under different N and irrigation management using different agronomic variables. Plant water provides a medium for enzymatic reactions and is also the raw material of photosynthesis. Plant water directly or indirectly affects the accumulation of plant material (Evans, 1989). In addition, LAI has a strong correlation with photosynthesis and respiration. Ata-Ul-Karim et al. (2014b) demonstrated that the N_c_ dilution curve as a function of LAI efficiently identified the N-limiting and non-N-limiting conditions. In addition, through the analysis of variance of multiple factors, different irrigation and fertilization treatments have a significant influence on LAI, but not on PWC. These results were similar to the results of significant differences of parameter “*a*” and parameter “*b*.” Therefore, it is feasible to express parameter “*a*” and parameter “*b*” using PWC and LAI, respectively. Both parameters are affected concurrently by water and N and show a decline ontogenically (Thornton et al., 1999), so they have a strong interaction with the critical dilution curve. However, in the present work they turned out to be useful for the improvement of the estimation of the mN_c_ dilution curve across years and experiments. Moreover LAI and PWC can be readily estimated from remote sensing (Ceccato et al., 2001; Upreti et al., 2019), it has great potential in promoting large-scale N status monitoring.

The accuracy of nitrogen diagnosis is vital to improve N use efficiency. The mN_c_ dilution model created in this study has more advantages in N diagnosis than standard LAI dilution model. In this paper, C_3_ crop, which has different N metabolism pathway and characteristics from C_4_ crop (Oaks, 1994; Bräutigam et al., 2014), the standard N_c_ dilution curves of C_3_ and C_4_ counstructed by Greenwood et al. (1990) also had significant differences. In order to improve N use efficiency, it is necessary to further construct mN_c_ dilution curve by combining N absorption, transport and signal transduction functions and regulatory mechanisms. This study was based on field-scale agronomic data for analysis and verification and did not address mN_c_ dilution curve over considerable areas. In order to establish a real-time, widely-applicable LAI diagnosis model, an appropriate N diagnosis model should be established under multiple cultivation modes, data sources, and scales.

## Conclusion

The accuracy of nitrogen diagnosis is vital to improve nitrogen utilization efficiency. It was shown that the standard LAI dilution curve could not be accurate across year under variable irrigation regimes. The PWC and LAI could be used to modify the standard N_c_ dilution curve by integrating the effect of water and N fertilizer. Under different management conditions, parameter “*a*” is more affected than “*b*.” The mN_c_ dilution curve considers different treatments and is represented by the equation mN_c_ = a×AGB^−*b*^, where *a* = 2.09 × PWC + 3.24, *b* = −0.02 × LAI + 0.51, with *R*^2^ and nRMSE of 0.79 and 0.13, respectively. We conclude that the N diagnostic approach developed in the present study provides a new procedure and an easy alternative tool to assess plant N status for guiding precision N management during the vegetative growth period.

## Data Availability Statement

The raw data supporting the conclusions of this article will be made available by the authors, without undue reservation.

## Author Contributions

YZ, ZL, and WY conceived, designed, and performed the experiments and analyzed the data. YZ, ZL, WY, PC, and GY discussed and drafted the manuscript. YZ, XX, JW, HF, and RC revised the manuscript and edited the English language. All authors read and approved the final version.

## Conflict of Interest

The authors declare that the research was conducted in the absence of any commercial or financial relationships that could be construed as a potential conflict of interest.

## Footnotes

1 http://www.ecmwf.int/
