# Supplementary figures and images for: A Modified Critical Nitrogen Dilution Curve for Winter Wheat to Diagnose Nitrogen Status Under Different Nitrogen and Irrigation Rates
Source: Front Plant Sci. 2020 Oct 21;11:549636. doi: 10.3389/fpls.2020.549636 (PMC7609434; doi:10.3389/fpls.2020.549636)

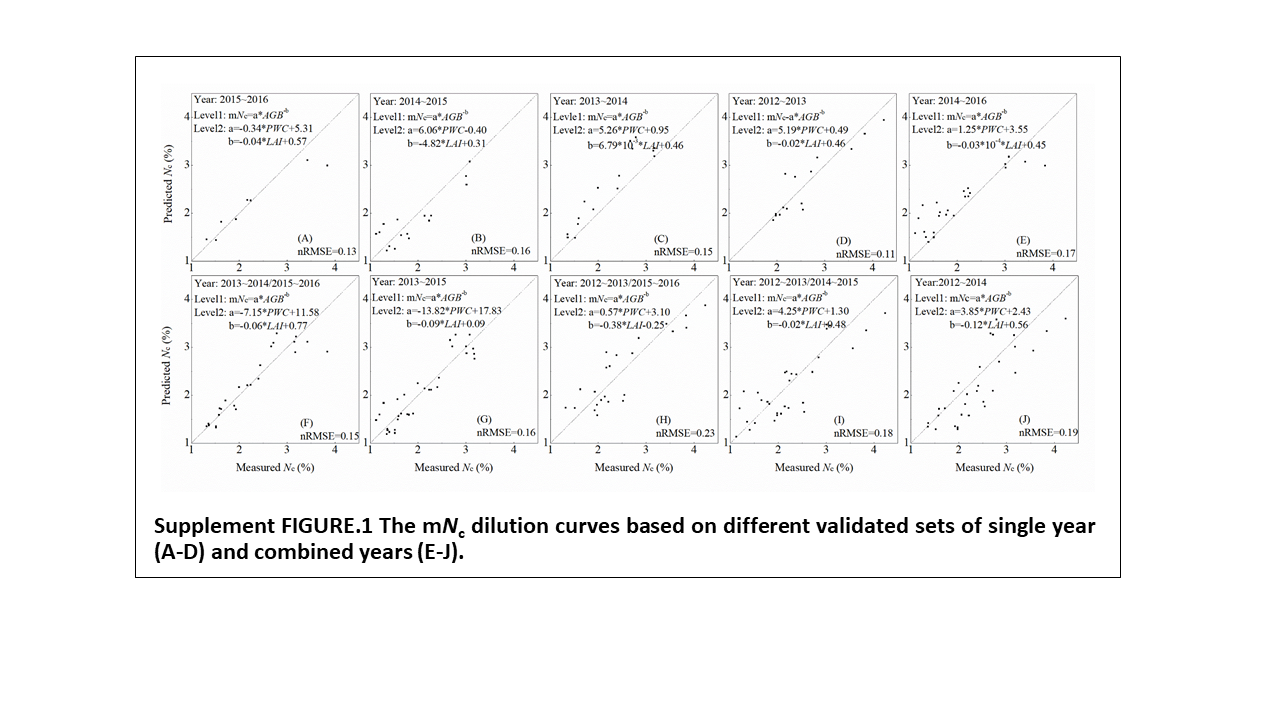

Supplement: Supplementary file 1 [file Image_1.TIF]
